# Gender affirming hormone therapy and transgender women fertility: Histologic predictors of germ cell presence

**DOI:** 10.61622/rbgo/2024rbgo33

**Published:** 2024-04-09

**Authors:** Lina Rigodanzo Marins, Tiago Elias Rosito, Lucia Maria Kliemann, Edson Capp, Helena von Eye Corleta

**Affiliations:** 1 Universidade Federal do Rio Grande do Sul Porto Alegre RS Brazil Universidade Federal do Rio Grande do Sul, Porto Alegre, RS, Brazil.

**Keywords:** Fertility, Hormone treatment, Spermatogenesis, Transgender women, Transgender persons, Fertility preservation, Gonadal steroid hormones

## Abstract

**Objective:**

Evaluate histological changes in testicular parameters after hormone treatment in transgender women.

**Methods:**

Cross-section study with patients who underwent gonadectomy at Hospital de Clínicas de Porto Alegre from 2011 to 2019. Hormone treatment type, route of administration, age at initiation and duration were recorded. Atrophy parameters were observed: testicular volume, tubular diameter, basal membrane length, presence of spermatogonia and spermatids (diploid and haploid spermatozoid precursors).

**Results:**

Eighty-six patients were included. Duration of hormone treatment is associated with testicular atrophy and spermatogenesis arrest. Other characteristics of hormone treatment such as age of initiation, route of administration and type of treatment were not associated with testicular histological changes. Testicular volume may predict spermatogenesis arrest. Basal membrane length and tubular diameter ratio is an interesting predictor of germ cell presence.

**Conclusion:**

Cross-sex hormone treatment affects testicular germ cell presence. Basal membrane length and tubular diameter ratio reduces inter variability of measurements and better exemplify how atrophic seminiferous tubules are. Fertility preservation should be addressed by healthcare providers in order to recognize gender affirming treatment impact on transgender health.

## Introduction

It is known that hormonal therapy in transgender improve significantly quality-of-life and psychosocial outcomes.^(1)^ However transgender individuals who undergo Gender Affirming Hormone Therapy (GAHT) may lose reproductive potential. Thus, before starting any treatment, patients should be encouraged to consider fertility issues.^(2)^ Most transgender patients are of reproductive age at the time of transition and 67% of young transgender women expressed a desire for future parenthood.^(3)^ Furthermore, 48% of transgender adolescents acknowledged that their desires regarding parenthood might change over time.^(4)^

Established fertility preservation methods exist for post pubertal males (sperm cryo- preservation), with experimental options available for younger children (for example: testicular tissue cryopreservation).^(5)^ Cryopreservation of surgically obtained spermatozoa through epididymal (PESA) or testicular sperm extraction (TESE) are alternatives for those who are unable to ejaculate or in case of azoospermia.^(6)^ These options are still underutilized by transgender patients and should be more explored and detailed.^(7)^

Transgender women using gender-affirming hormonal medication at the time of fertility preservation have abnormal semen parameters and may be azoospermic, especially when the ejaculate volume is low.^(8)^ Decreased diameter of seminiferous tubules and expansion of the interstitium, hypoplasia or absence of Leydig cells, and peri epididymal fibrosis are described and may explain the low sperm quality found.^(9)^ Determinants of hormone therapy such as timing of initiation, duration of treatment, type and route of administration are not well studied regarding their impact on spermatogenesis.^(10)^ Discontinuation of gender-affirming medication may be associated with an improvement in semen parameters, however long-term studies are lacking.^(9)^

In order to offer fertility preservation, it is important to determine how does gender affirming therapy affects testicular histology in transgender women. The aim of this study is to evaluate the impact of different hormonal therapies on the testicular histology of orchiectomies from gender-affirming surgeries. A ratio of basal membrane over tubular diameter is proposed as a predictor of germ cell presence.

## Methods

Cross section study with histological analysis.

This research is part of PROTIG initiative (gender identity disorders program) held in Hospital de Clínicas de Porto Alegre from 2011 to 2019.

All participants included were enrolled in the program and were followed at least two years before gender affirming surgery. A multidisciplinary time is formed by endocrinologists, urologists, psychologists, psychiatrists, gynecologists, breast surgeons and nurses with periodical follow up. Participants who underwent gender affirming surgery during 2011 to 2019 were included. Charts were reviewed to assess hormone treatment prescriptions and use, demographic and health status data. Data collection: Gender Affirming Hormone Therapy (GAHT).

Information was obtained by medical chart review. Standard hormone treatment included oral estrogen and cyproterone or spironolactone regimens. Dosage was adjusted according to clinical parameters and participant satisfaction. Previous hormone regimens were described in personal charts regarding dosage, timing of initiation, route of administration, duration and regularity of use.

All participants underwent gender affirming surgery at Hospital de Clínicas de Porto Alegre. Orquiectomy specimens were fixed in formalin and embedded in paraffin. The samples were sectioned and stained in hematoxylin-eosin.

**Figure 1:**
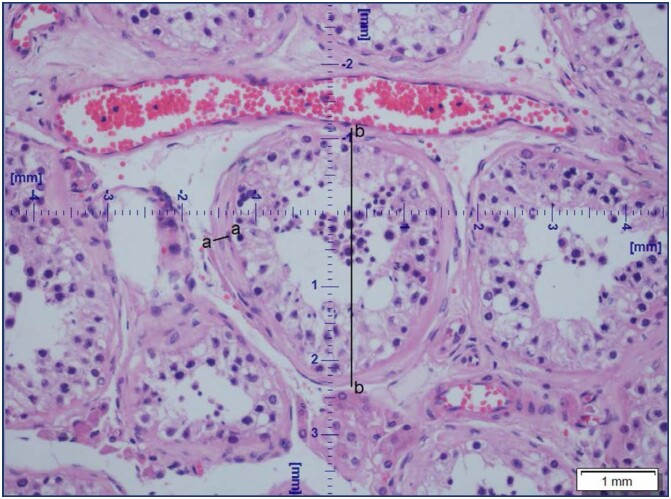
Measurement of tubular diameter(b-b) and basal membrane length(a-a)

The stained sections were analyzed by using an optical microscope (Olympus LX80). Pictures of the sections were taken with CELL F imaging software (Life Science Technology). Images were calibrated at a magnification of X20, and cross-sections of seminiferous tubules that were either round or nearly round were selected by an experienced pathologist, who took three pictures from each sample where at least one cross-sectional view of seminiferous tubules should be present from a different region of the testis. The diameter of the seminiferous tubule diameter and basal membrane thickness were measured directly in the CELL F imaging software (Life Science Technology), in at least five different tubules per patient across the minor and major axes, and the mean diameter was obtained and reported in micrometers (Figure 1). Basal membrane thickness and tubular diameter ratio then transformed into percentage in order to favor interpretation. Maximum value of MB/TD is 50%.

The presence or absence of spermatogonia (diploid form) and spermatids (haploid form) was examined by an experienced pathologist in up to 10 different tubules.

Data were analyzed using IBM SPSS 25.0 (SPSS, Chicago, IL, USA). Normal distribution was verified using Shapiro-Wilk test. Normal data are shown as mean ± standard deviation (SD), and non-normally distributed values are shown as medians (percentile 25 to percentile 75). For normally distributed values, Pearson's test was used, for non-normally distributed values, Spearman's Rho. Non-normally distributed variables were compared among groups using Mann–Whitney U test and Kuskall Wallis test. Differences in frequencies between groups were compared using chi-squared statistic. In case of low cell count, differences in frequencies were compared using Fisher's exact test. The significance level was set at P<0.05.

Ethical approval was given by Hospital de Clínicas Ethical Committee (3.695.060). CAAE: 18408819.2.0000.5327.

## Results

Our cross-section study included 86 transgender women submitted to gender affirming surgery, 21 to 59 years old, median of 35 years at gender affirming surgery. Population description is available in table 1. Markers of social vulnerability are remarkably high among our study population 62% presented psychiatric disorders (diagnosis carried out by psychiatry and psychology professionals from the multidisciplinary team), 31% have a history of sexual abuse and 31% are smokers. Once enrolled in the PROTIG program, participants were closely followed and they could only proceed with affirmative surgery after two years participating in group and individual meetings.

**Table 1:** Age at surgery, hormonal treatment characterization, and histological findings

Parameter	Median	Minimum	Maximum
Age at surgery (years-old)	34.86	21	59
Age of starting GAHT (years-old)	21	14	51
GAHT duration (years)	7.5	1	34
Testicular volume (cm^3^)	11.7	4	51.9
Seminiferous tubular diameter (TD, µm)	162.1	73.6	967.4
Basal membrane (BM, µm)	23.8	5.5	278.53
BM/TD-ratio	10.9	3.2	50.00

GAHT - gender affirming hormone treatment; BM/TD ratio - basal membrane, seminiferous tubular diameter ratio x100

Hormonal treatment characteristics, testicular volume and histological findings are shown in table 2. Minimum age at surgery was 21 years old and hormonal treatment duration was in average 7.5 years, with minimum treatment duration of 1 year. Testicular volume varies from 4 to 51 (cm^3^). The mean BM/TD ratio x 100 was 10.9.

**Table 2:** Gender affirming hormone tratment prescribed: route of administration and medical compounds

Parameter		n(%)
GAHT route of administration		
	Oral	47(54.7)
	Parenteral	16(18.6)
	Both	23(26.7)
Medical compound		
	Estrogen + cyproterone or spironolactone	39(45.3)
	Estrogen+progestogen	17(19.8)
	Estrogen only	30(34.9)

GAHT - gender affirming hormone treatment, values expressed in absolute number (percentages)

As shown in table 2, oral GAHT was preferable route of administration (54,7%), but 26% of the patients have used oral and parenteral hormone therapy. Different estrogens (conjugated estrogens, estradiol valerate, ethinyl estradiol) plus medications with anti-androgens properties, such as cyproterone and spironolactone were the majority of prescriptions as GAHT for transgender woman (45%), 35% used only estrogen and 20% combination of estrogens and progestogen. Regarding germ cell presence, diploid form (spermatogonia) is present in the majority of cases (70.9%, n= 61) meanwhile, haploid form, spermatid, is only shown in 37.2% (n= 32) of patients. In 29,1% of patients no germ cell was not found. All variables analyzed were non-parametric according to the Shapiro-Wilk test. Correlation analysis are presented in tables 3 and 4.

**Table 3:** Parameters of gender affirming treatment and fibrosis markers: how does it impact on germ cells presence?

Parameters	Spermatogonia presence	Spermatids presence
Age of initiation GAHT	0.355 (0.001)*	0.225 (0.037)*
Duration GAHT	0.005 (0.962)	0.067 (0.543)
Testicular volume	0.238 (0.027)*	0.274 (0.011)*
Tubular diameter (TD)	0.315 (0.003)*	0.306 (0.004)*
Basement membrane (BM)	-0.581 (<0.001)*	-0.409 (<0.001)*
BM/TD ratio	- 0.679 (<0.001)*	-0.578 (<0.001)*

* Values display Sperman's correlations amongst variables described and its p-value; Significance is considered with p<0.05, marked with

**Table 4:** Hormone treatment parameters and testicular histologic markers

Hormone treatment parameters		Tubular diameter (TD)	Basement membrane (BM)	BM/TD Ratio
Route of administration				
	Oral	195.23 (135.94-266.13)	21.41 (11.14-37.64)	8.66 (5.62-22.72)
	Intramuscular	133.35 (127.00-181.03)	20.50 (12.62-38.01)	10.57 (6.32-27.22)
	Both	127.04 (106.69-247.23)	41.74 (13.09-53.50)	21.64 (7.86-41.17)
P^a^		0.079	0.195	0.049
Type of hormone therapy				
	Only Estrogen	171.47 (129.40-243.42)	18.61 (11.26-45.39)	9.26 (6.24-34.04)
	Estrogen and antiandrogen	165.32 (125.61-317.71)	26.26 (10.20-42.73)	10.80 (5.62-26.92)
	Combined	127.31 (115.35-198.94)	24.22 (12.16-40.44)	11.53 (7.10-26.08)
P^a^		0.373	0.986	0.818
Age initiation hormone therapy^b^		0.106 (0.334)	-0.034 (0.756)	-0.095 (0.382)
Duration of hormone therapy^b^		-0.149 (0.170)	0.249 (0.021)	0.295 (0.006)

a Test of Kruskal-Wallis, data presented as "median (QI25 and QI75)";

b Spearman's correlation, data presented as "rho (P)"

Hormone treatment parameters have different impacts in fibrosis markers as shown in table 4. Age of hormone therapy initiation was positively correlated with germ cell presence however didn't achieve statistical correlation with fibrosis’ parameters. Similarly, type of GAHT does not correlate with atrophy observations. Route of administration (oral, parenteral or both) and BM/TD ratio was significantly correlated. GAHT duration is correlated with BM/TD ratio positively and negatively with germ cell's presence.

**Figure 2:**
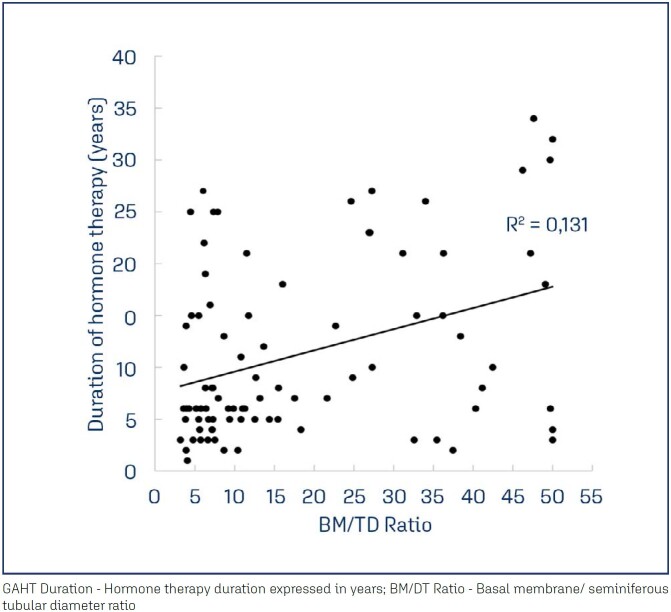
Correlation of hormone treatment duration and testicular fibrosis marker

Hormone treatment duration and basal membrane/tubular diameter ratio correlation is shown in figure 2.

## Discussion

There are several theoretical mechanisms of how testosterone alterations and estrogen exposure impair spermatogenesis. Compromise in integrity of blood-testis-barrier with increased vulnerability to cytotoxic and immune damage; impaired adhesion with premature detachment of spermatids from Sertoli cells, causing altered shaping in spermatids; germ cells phagocitation.^(11)^

All these mechanisms can lead to impaired semen quality observed in transgender women, with lower total sperm count.^(12)^ At the time of fertility preservation only 26.4% of the post-thawed samples showed adequate semen quality for intrauterine insemination.^(10)^ Samples revealed a high incidence of azoospermia, oligozoospermia and asthenozoospermia, despite known factors associated with decreased semen quality in the general population (smoking, BMI, others).^(13)^ Lower dosages of ethinylestradiol (20 micrograms/day) were not associated with impaired motility or density, while 60 micrograms per day were, showing a dose dependent mechanism.^(4)^

However, some studies show that even before GAHT, transgender women already demonstrate altered sperm quality. Tucking, low masturbation frequency and wearing tight underwear are suggested explanations.^(10)^

Testicular volume seems to predict spermatogenesis with a positive correlation, as seen in our results. Schneider et al., in 2015,^(14)^ associated a 25% reduction in volume of testis with depletion of germ cells. This decline in testis weight represents a valid readout of the efficiency of spermatogenic suppression.^(14)^ This finding is seen in other studies.^(12,15)^ However, we could not correlate testicular volume with fibrosis parameters, suggesting that spermatogenesis arrest may occur earlier than hyalinization and reduction in seminiferous tubular diameter. On the contrary, a higher basal membrane/tubular diameter ratio is associated with spermatogenesis arrest as seen in table 3.

Previous histological analyses of orchiectomies are described in literature. Maturation arrest was found in 36.4%, hypospermatogenesis in 26%, Sertoli cell-only syndrome in 20.2%, normal spermatogenesis in 11%, and seminiferous tubule hyalinization in 6.4% of the specimens.^(15)^ Matoso et al.^(9)^ describe decreased diameter of seminiferous tubules and expansion of the interstitium, marked hypoplasia of germ cells, rare cytomegaly, hypoplasia or absence of Leydig cells, and epididymal hyperplasia. Testicular neoplasia was not found in any of case studies in more than 2500 trans women submitted to orquidectomy.^(16)^

Our results showed reduced tubular diameter and increased basal membrane length, pointing out towards a pattern of fibrosis and hyalinization of testis overtime regarding GAHT duration. Other studies could not find an association between GAHT duration and altered histological findings. Majority of these studies had a lower mean time duration of therapy and smaller samples.^(9,17)^ A role of GAHT age of initiation is proposed, since complete spermatogenesis occurs in Tanner stage 3 and onwards.^(18)^ We could not find this association, but only a minority of our patients initiated GAHT in pre-pubertal stage. Regarding spermatogenesis, majority of patients still exhibited germ cells levels-spermatogonias or spermatids. Therefore, our data is in agreement in what is already known in literature: despite long-term hormone therapy, majority of transgender women have germ cells present in testicle. Spermatogenesis is preserved in approximately 40% of these individuals. However, spermatozoids were not seen in our samples and represent a final step of gametogenesis.

Regarding other GAHT parameters, such as dose, route of administration and medical compound prescribed, data are scarce and conflicting. Majority of studies use an estrogen only regimen and few describe anti androgen medication.^(18)^ We could not find differences between our groups, probably due to sample size. A trend towards increased lamina propria length/ reduced tubular diameter ratio and parenteral or mixed (oral and parenteral) administration of GAHT was seen.

BM/TD ratio is an interesting marker of germ cell presence and it has not been previously described. Previous study used isolated tubular diameter and thickness of tubular wall as predictors of sperm Retrieval.^(19)^ However, BM/TD ratio was designed to minimize inter-tubule changing parameters upon observation. This ratio also provides an instrument of easier interpretation, since it represents the percentage of diameter of the tubule that is filled with basal membrane, and hence atrophic. It showed a strong negative correlation with germ cell presence and a positive correlation with GAHT duration.

Spermatogenesis arrest may be reversible with GAHT cessation, although data are conflicting in literature.^(8)^ If premenopausal female hormone levels are targeted, complete suppression of spermatogenesis is expected.^(17)^ If not, anticonception advice should be offered due to a theoretical risk of unplanned pregnancy. Interestingly, after fertility preservation techniques with surgically obtained spermatozoa pregnancy rate per cycle has been reported to be 22.8% and the live birth rate 22.3%.^(18)^

We recognize some study limitations. A single center was enrolled and the population may differ from previous studies. It is a cross-sectional study without matching controls, so causality cannot be inferred.

## Conclusion

Specific estrogen regimens varied widely among patients and serum hormonal levels were not monitored. We emphasize that histologic examination of the testicle is only a preliminary avenue of study and its true outcome regarding fertility is theoretical. Nonetheless, since data are scarce in this subset of patients, we acknowledge the importance of our contribution in the better understanding of future perspectives and patient care in transgender health. To our knowledge, this is the first study that evaluated the influence of other GAHT parameters in testicular histology and proposed BM/TD ratio as a predictor of germ cell presence.
